# The role of *EZH2* dysregulation in the pathogenesis of B-cell lymphomas and its implications as a target therapy

**DOI:** 10.3389/fonc.2026.1843273

**Published:** 2026-05-13

**Authors:** Myrna Candelaria

**Affiliations:** 1 Hospital Angeles Pedregal, Mexico City, Mexico; 2 Instituto Nacional de Cancerología México, Mexico City, Mexico

**Keywords:** B-cell lymphoma, diffuse large B cell lymphoma, epigenetics, EZH2, follicular lymphoma, non-Hodgkin lymphoma

## Abstract

B-cell lymphomas are a group of heterogeneic diseases. During B-cell development, the germinal center (GC) reaction is a complex process, in which transcripcional programs have an epigenetic regulation. Enhancer of zeste homologue-2 (EZH2) is a histone methyltransferase, which catalyzes trimethylation of histone H3 at lysine 27 (H3K27me3) to control gene transcription critical for cell proliferation, function, differentiation and expansion. It controls the normal biology of germinal B cells. Epigenetic dysregulation is a hallmark of Diffuse large B- cell lymphoma (DLBCL) and follicular lymphoma (FL), with gain of function (GOF) *EZH2* mutations. EZH2 is also overexpressed and plays an important role in lymphomagenesis. Follicular lymphoma (FL) pathogenesis includes epigenomic alterations affecting the immunological niche, and their potential consequences on the informational transfer between the microenvironment and tumor B cells. The introduction of novel target therapies and immunotherapeutics modalities in B cell lymphomas has shifted the treatment landscape. Tazemetostat received FDA approval as the first epigenetic therapy for FL. Valemetostat, which inhibits EZH1/2, as well as tazemetostat in combination with other drugs have been subject of recent research. The purpose of this article is to review the role of EZH2 in normal B cell differentiation and in the pathogenesis of B-Cell lymphomas.

## Introduction

Non- Hodgkin lymphoma (NHLs) is a group of heterogenous malignancies derived from T & NK cells (about 10%) or B lymphocytes (approximately 90%) (1).

It is the seventh most prevalent cancer in the United States and has the sixth-highest mortality rate (2). Mature B-cell neoplasms are a diverse group arising from antigen-experienced B-Cells. The most frequent B- cell lymphoma is the Diffuse large B-Cell lymphoma (DLBCL) (30%), followed by follicular lymphoma (FL) (25%), mucosa-associated lymphatic tissue (MALT) lymphoma (7%), chronic lymphocytic leukemia/small lymphocytic lymphoma (CLL/SLL) (7%), and mantle cell lymphoma (MCL) (5%) (3, 4).

DLBCL has a heterogeneous genetic and epigenetic landscape, which influences in the immunophenotype and therapy response.

FL is originated from B cells with the t(14;18) translocation. It constitutes a disease driven by epigenetic dysregulations with mutation in the genes encoding for histone-modifying enzymes identified as early initiating drivers, as EZH2, CREBBP and KMT2D. (5).

The advances in the understanding of pathogenic mechanisms implicated in lymphomagenesis have allowed the development and design of several anticancer drugs, including epigenetic therapies as: EZH2 inhibitors, DNA methyltransferases (DNMTs), inhibitors of histone deacetylases (HDACs), bromo-domain and extra-terminal domain proteins (BETs), isocitrato deshydrogenases (IDHs), and protein arginin N-methyltransferases (PRMTs).

## Homolog 2 of enhancer of Zeste

The *EZH2* gene is evolutionarily conserved from drosophila to mammals (6). It is located on 7q36.1, within the exon 25. It encodes a member of the polycomb family, which is responsible for the transcriptional repressive state of genes. PRC2 has four components: Zeste 2 polycomb repressive complex 2 subunits (EZH2), EED, SUZ12 and RbAp48 (7). PRC2 acts upstream of PRC1 as H3K27me3 serves as a “docking” site for CBX, a chromodomain containing protein, which recruits PRC1 to induce H2AK119ub1. EED also interacts with PRC1, and links PRC2 and PRC1 (8, 9). The catalytic core of PRC2 is located at the C-terminal end of the conservative domain of *EZH2* (4, 8, 10).

EZH2 protein contains a C-terminal catalytic SET-domain, an adjacent CXC domain, two SANT domains, and an in-between ncRBD domain. The SET and CXC domains are required for histone methyltransferase activity (11). The SANT domains enable EZH2 to bind to DNA for chromatin remodeling and transcriptional regulation (12); the ncRBD domain is the binding region with non-coding RNAs (6).

EZH2 catalyzes 1 to 3 methylations of Lys27 on histone H3, which represses gene transcription (13). Wild-type EZH2 catalyzes the mono-and dimethylation of H3K27, whereas the mutated EZH2^Y646^ produces an enzyme that primarly catalyzes the third methylation (14, 15).

## Epigenetic mechanisms in B-cell maduration

B-cell maturation is a complex process regulated by the interplay of different stage-specific TFs and epigenetic complexes. The four main epigenetic modifications include DNA methylation, histone modifications, chromatin remodeling by epigenetic regulators and noncodign RNAs (3, 16-18).

DNA methylation: It is associated with gene silencing and involves the addition of a methyl group to cytosines at CpG dinucleotides, which are located in the promoters and/or first exons of genes (17). DNMT3A and DNMT3B enzymes are responsible for *de novo* methylation. During the transit of centroblasts, the DNMT3A expresión is downregulated. In contrast DNMT1 is upregulated (3).

Histone modifications: Histone acetyltransferases add an acetyl group, and it is removed by the histone deacetylases (HDACs). Acetylation is frequently associated with gene expression, and it takes place at the H3K9, H3K18, H3K27, H3K4, and H3K5 lysine residues. On the other side, histone methylation is mediated by methyltransferases and it occurs at H3K4 (by MLL1-5, SET1-A, SET1-B), H3K9 (by SUV39H1), H3K27 (EZH2), H3K36 (SET2, NSD1), and H3K79 (DOT1-L) (19).

EZH2 represses the expression of genes that are transcribed in naive B cells (pre-B cells) (20). EZH2 allows the optimal V(D)J recombination in the adult bone marrow, and represses genes involved in cell cycle arrest and terminal differentiation. EZH2 levels are low during early B cell stages but increase at the time of GC transit.

Chromatin remodeling: B-cell maturation depends on chromatin remodeling for its regulation, especially in the GC where activated B cells undergo clonal expansion, somatic hypermutation, and class-switch recombination (21). The subunit BRG1 from the SWI/SNF (BAF) complex has a crucial role, driving B-cell activation and GC development (Schmiedel et al., 2021). The H1 mutations lead the chromatin decompactation and enable B-cells to self-renewal, disrupting the chromatin organization (22).

Non coding RNA: The non-coding RNA act as guides, signals, or scaffolds modulating gene expression at transcriptional and post-transcriptional levels. They control gene expression during B cell maturation through their action at developmental checkpoints (23).

The germinal center (GC) has two regions: The dark zone (DZ) with very proliferative B cells, in which immunoglobuline (Ig) class-switch recombination and somatic hypermutation (SHM) take place, and the light zone (LZ), where non-dividing GC B-cells interact with follicular dendritic cells to receive help for cycling into the DZ or maturation into plasma cells (PCs) (24, 25).

EZH2 impacts B-cell lineage commitment, maintenance, and transition through governing a repertoire of silent genomic loci (6). The level of EZH2 is abundantly seen in pro-B cells, thereafter it drops drastically to pre-B cells (20, 26). Proliferating GC B-cells (centroblasts) undergo to somatic hypermutation due to the action of the activation induced cytidine deaminase (AICDA). This process allows the diversification of immunoglobulin genes within the GC. After several rounds of replication, these cells migrate to the light zone, which contains specialized T cells called T-follicular helper (T_FH_), as well as folicular dendritic cells (20, 27–30).

When resting B cells enter into cell cycle, H3K27 tri-methylation increases along with other histone H3 methylation forms to establish B cell identity at different stages (31). In this process EZH2 silences anti-proliferative genes, including CDKN family, such as *CDKN1A* and *CDKN1B* (25). The silencing of these genes occurs in cooperation with BCL6 and BCOR (32). It also marks provisions for antibody diversification, affinity maturation and secretion (33, 34).

EZH2 also plays a role in many processes within the microenvironment, including CD4+, NK cells and CD8 T differentiation and functions (13).

## The role of EZH2 in lymphomagenesis

Mutations in chromatin-modifying enzymes, as EZH2, CREBBP, KMT2D or TET2 disrupt normal gene expression programs essential for B cell maturation and facilitate the malignant transformation (3, 35).

*EZH2* mutations were primarly observed in FL and the GC group of DLBCL, affirming they share molecular underprintings (15). These mutations have detrimental consequences on the anti-tumor immune response in B-cell malignancies (14, 36).

Gain-of-function mutations of *EZH2*, are located most commonly at the Tyr641 residue within the SET domain (Y641H, Y641S, Y641 C, and Y641N) (6, 37) They allow the conversion of mono- or di-methylated H3K27 to the tri-methylated state, promoting the repression of cell differentiation and down-regulation of tumor suppressor genes (38), which drive germinal center hyperplasia and help to remodel the immune microenvironment (14, 38), a process which supports the activity of T cells toward GC-B cell development, and is ensured by folicular dendritic cells (FDCs) (39). Additionally, EZH2 overexpression has been associated with a poor prognosis (40) in patients with B-cell lymphomas.

B-cells with *EZH2* mutations result in epigenetic silencing, B cell proliferation and clonal development of malignant cells. They are also associated with MYC lymphomagenesis in B cells (8, 41).

EZH2 can also reprogram malignant cells to affect interactions with the microenvironment (15).

These *EZH2* mutations are found in 6-20% of DLBCL overall (37, 42) and are frequently associated with BCL2 translocations (38). These mutations are heterozygous and cause massive expansion of GC, a classical manifestation of oncogenes that drive lymphomagenesis (43). Additionally, other genetic lesions of *EZH2*, including copy number amplification, chromosomal gain or loss, and DNA hypermethylation have also been identified in DLBCL (44) or FL (35).

*EZH2*^Y641^ GC B-cells don´t depend on T cells to maintain survival and proliferation (45).

EZH2 and transcription regulators IKZF1 and IKZF3 share target genes, including *IRF4*, which suggest a synergy between EZH2 and these cereblon targeting agents (46). The addition of H3K27me3 at gene promoters, leads the silencing of the *IRF4* and *PRMD1* genes involved in plasma cell differentiation. BCL6, a transcriptional repressor, cooperates with EZH2 in GC-cells, recruiting SMRT and NCOR, which mediates H3K27 deacetylation. This process promotes SMRT phosphorilation and the exit of B cells from GC (47) The MHCII deficiency leads to escape from immune surveillance and inferior prognosis (48).

Additionally, *EZH2* activating mutations contribute to both MHCI and MHC II repression (48). EZH2 activity impacts on cell metabolism, but also cell metabolism impacts on EZH2 function (31). Overexpressed EZH2 hypermethylates the promoter of FAS-AS1, leading to impaired FAS-mediated apoptosis (49). EZH2 also represses the tumor-suppressive BLIMP-1 in GC-B cell lymphoma (50).

EZH2 may coordinate with other oncogenic regulators (31). Also, EZH2 is involved in the maintenance of EBV latency and reactivation. Both, EZH2 and EBV, synergize to silence tumor suppressor genes to drive B cell lymphomagenesis (6, 51).

cfDNA can detect genetic alterations in different subtypes of DLBCL, which makes a helpful tool for disease genotyping, and allows the molecular classification of DLBCL (52). In particular, regarding *EZH2* mutations, Diaz-Chavez et al. demonstrated the feasibility of documentation of *EZH2* Tyr 641 mutations (Y641 F, Y641 N, Y641S, and Y641H) in cfDNA and the negative impact on prognosis in patients with these mutations (53).

In follicular lymphoma, also the gain-of-function *EZH2* mutations occur in 20- 25% of cases. They are among the first epigenetic alterations, and constitute critical drivers in the early stages of FL. As a key epigenetic writer, EZH2 silences regulators of genes involved in cell cycle checkpoints, differentiation, and immune synapse signaling, reducing the dependency of GC cells on T cells (43, 54). *EZH2* mutations also promote the expansion of centrocyte-like population characterized by proliferation and survival.

Dysregulated signaling pathways also cause EZH2 overexpression. C-MYC overexpression or rearrengements upregulate EZH2 through repression of the EZH2-targeting miRNAs (mir-26a, mir-26b, mir-101) and EZH2 induces MYC expression via inhibition of the MYC targeting miR-494 (41, 55).

The interplay between EZH2 and IncRNA has a role in oncogenesis. In DLBCL, upregulated HOTAIR is validated as an indicator of poor prognosis, and a possible mechanism for inducing H3K27 trimethylation via EZH2 related PRC2 inactivation (56). MALAT1 also regulates cancer cell metastasis (57) and chemoresistance in different tumors.

Postranslational modifications of EZH2, mainly phosphorylation, also influence in a critical step of oncogénesis (6, 58).

A deeper understanding of the epigenetic alterations governing B-cell malignancies are promising potential needed to refine the prognostic models, to improve the risk stratification scores and allow new therapeutic approaches that may induce durable remissions.

## Therapeutics

EZH2 has emerged as an attractive therapeutic strategy because its noncytotoxic mechanism of action (59).

Among EZH2 inhibitors, tazemetostat has recently emerged, as an oral inhibitor, which reduces the methyltransferase activity in both *EZH2* mutant and wild-type. The phase I trial included relapsed/refractory B-cell NHL (n=21) or advanced solid tumors (n= 43), with increasing doses from 100 twice daily up to 1600 mg twice daily in 28-day cycles. The dose-limiting toxicity was grade-4 thrombocytopenia at the dose of 1600 mg twice daily. Other grade 1–2 adverse events were asthenia (33%), anorexia (6%), muscle spasms (14%), nausea & vomiting (9%), and anemia (14%). The recommended dose was 800 mg twice daily.

The phase II trial included relapsed/refractory follicular lymphoma patients, from them, 45% had *EZH2* mutations. The overall response rate (ORR) and complete response (CR) rate was 69% & 13%, respectively in mutant *EZH2*. In the wild-type *EZH2* patients, the ORR & CR was 35% and 4%, with a median progression free survival (PFS) of 13.2 months. Most common adverse events included nausea, diarrhea, alopecia, and fatigue.

Since a higher response rate is documented in *EZH2* mutated lymphomas, the testing for *EZH2* mutations in patients with FL after at least 2 lines of treatment, is recommended in the United States National Comprehensive Cancer Network (NCCN) guidelines (60).

Clinical trials combining tazemetostat and other drugs have been developed and are ongoing (**Table 1**) (61).

**Table 1:** Ongoing clinical trials combining tazemetostat and other drugs (41).

Agent(s)	Trial name (phase)	Comments
Frontline setting -FL		
Tazemetostat +RCHOP (induction) followed by maintenance tazemetostat for 6 months and rituximab for 2 years	NCT02889523 (Phase III)	High-grade FL
Tazemetostat and Mosunetuzumab in Untreated Follicular lymphoma	NCT05994235 Phase II	FL
Tazemetostat + RCHOP for 6 cycles	NCT02889523 Phase I/II	DLBCL
Relapsed/Refractory setting		
Tazemetostat/Venetoclax in Non-Hodgkin lymphoma	NCT05618366 Phase 1, single arm	DLBCL & FL
Tazemetostat plus CART cell therapy in B cell lymphomas	NCT05934838 Feasibility trial.	FL, B cell lymphoma. Mantle cell lymphoma
Tazemetostat in combination with zanubrutinib and anti-CD20 monoclonal antibody in relapsed or refractory B cell Non-Hodgkin lymphoma	NCT06824701 Phase 1B	B-cell non Hodgkin lymphoma.
A study to assess the efficacy, safety, pharmakodynamics and pharmakokinetics of tazemetostat in combination with lenalidomide plus rituximab versus placebo in combination with lenalidomide plus rituximab in Adult patients at least 18 years of age with relapsed/refractory Follicular lymphoma	NCT04224493 Phase 1b	FL
Pembrolizumab and tazemetostat to Overcome immune tolerance Following ASCT or CAR-T cell therapy in patients with Agressive B-cell Non Hodgkin Lymphoma	NCT06242834 Phase II	B-cell non Hodgkin lymphoma
Tasting the safety and anti-cancer Drugs tazemetostat and belinostat in patients with lymphomas that have Resisted Treatment	NCT05627245 Phase I.	Recurrent B-cell Non Hodgkin lymphoma. Recurrent FL and DLBCL
Study adding drugs to usual treatment for Large B-cell lymphoma that Returned or Did Not Respond to Treatment.	NCT 05890352 Phase II	Grade 3b folllicular lymphoma. High grade B-cell lymphoma High grade B-cell with MYC and BCL2 and/or BCL6 rearrengements.
Study combining tazemetostat with umbralisib and ubituximab in heavily pretreated patients	NCT05152459	Follicular lymphoma. This trial explores the sinergy between targeting EZH2 (epigenetic regulator) and Pi3K (signaling pathway) to improve outcomes in heavily pretreated patients.

Possible mechanisms of tazemetostat resistance have been evaluated in DLBCL cell lines and include overactivation of the phosphatidylinositol 3-kinase (PI3K) and mitogen-activated protein kinase (MAPK) pathways (50). Recently, a clinical trial is exploring the sinergistic effect of the combination of tazemetostat with inhibitor of the Pi3k pathway in heavily pretreated patients with FL (see **Table 1**). However, combined inhibition of EZH2 and PI3Kδ significantly increased the frequency of chromosomal translocations, surpassing the effects of EZH2 and PI3Kδ inhibition alone.

Valemetostat, an EZH1/2 inhibitor, has been evaluated in clinical trials including B- and T-NHL subtypes (NCT02732275). The ORR was 53%, most of them were T-cell lymphomas (ORR 80%).

## Conclusions

Although DLBCL &FL have been classified in different entities, they share pathogenic pathways, including the epigenetic regulation through EZH2. GOF *EZH2* mutations have a role in the pathogenesis, and a negative impact on prognosis of both lymphomas. Therefore, new drugs have been developed targeting this epigenetic regulator. However, the interaction between them and possible secondary deleterious effects require to be confirmed, as the combination of tazemetostat with PI3k inhibitors, which increased the frequency of chromosomal alterations (62).
